# The use of virtual reality computed tomography simulation within a medical imaging and a radiation therapy undergraduate programme

**DOI:** 10.1002/jmrs.436

**Published:** 2020-09-30

**Authors:** Therese Gunn, Pamela Rowntree, Deborah Starkey, Lisa Nissen

**Affiliations:** ^1^ School of Clinical Sciences Faculty of Health Queensland University of Technology (QUT) Brisbane QLD Australia; ^2^ Institute of Health and Biomedical Innovation (QUT) Brisbane QLD Australia

**Keywords:** Education, computed tomography, medical imaging, radiation therapy, virtual reality

## Abstract

**Introduction:**

The use of virtual reality (VR) simulation in the education of healthcare professionals has expanded into the field of medical radiation sciences. The purpose of this research was to report on the student experience of the integration of VR education for both medical imaging (MI) and radiation therapy (RT) students in learning computed tomography (CT) scanning.

**Methods:**

A survey was performed to evaluate students’ perceived confidence in performing diagnostic and planning CT scans in the clinical environment following VR CT simulation tutorials. Students from both MI and RT participated in providing quantitative and qualitative data.

**Results:**

The MI students (*n* = 28) and RT students (*n* = 38) provided quantitative results linking their engagement (perceived usefulness, ease of use, enjoyment) with their perceived confidence. The 15 (54%) MI students who recorded a maximum engagement score had a mean confidence score 1.02 higher than the students not fully engaged (Fisher’s exact test 14.549, *P* = 0.00). The results from the RT cohort revealed 68% of students agreed or strongly agreed to the addition of VR CT simulation helping in the learning of CT.

**Conclusion:**

It can be concluded that the integration of innovative learning opportunities such as VR CT simulation has the potential to increase student confidence and improve student preparation for the clinical environment.

## Graphical abstract

The purpose of this research was to investigate the integration of virtual reality education for both medical imaging and radiation therapy students in learning CT scanning. Students' learning can be optimised by repetitive practice in a safe environment and can be associated with increase student clinical confidence. A more confident student has the potential to improve student efficiencies in the clinical environment.

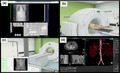

## Introduction

Simulation in health care has been used as a successful training tool to improve student skill and confidence in many disciplines, including nursing^1^ and medical radiation sciences (MRS).^2^ As digital technologies have advanced within society, the inclusion of virtual reality (VR) simulation for healthcare training has increased.

The use of VR to aid in the learning of radiation therapy (RT) students has been well documented and evaluated using the Virtual Environment Radiotherapy Training (VERT^©^)^3^ system.^4^, ^5^, ^6^ Literature on the use of VR simulation to aid the general radiographic skills of medical imaging (MI) students is slowly gaining momentum.^7^, ^8^ Shanahan reported on the value of virtual simulation as an educational tool to support pre‐clinical skill development in MI.^9^

### Virtual reality simulation

When defining the term virtual reality simulation, Merriam and Webster^10^, ^11^ provide a breakdown of the two components:

Virtual Reality – *‘an artificial environment which is experienced through sensory stimuli (such as sights and sounds) provided by a computer and in which one's actions partially determine what happens in the environment’*.

Simulation – *‘examination of a problem often not subject to direct experimentation by means of a simulating device’*. Where simulating is defined as *‘to give or assume the appearance or effect of’*.

VR has been documented to effectively train high‐risk professions such as commercial pilots and military personnel.^12^, ^13^ The stated reasons for VR simulation success are as follows: it enables users to focus on specific tasks; it can present uncommon or serious scenarios; students can engage in self‐directed learning; mistakes can provide learning opportunities without negative impact; students are motivated; students restricted by distance or location can engage in learning activities.^12^, ^13^, ^14^

Some reported barriers for the use of virtual environments within higher education have included challenges using technology and institutional and personal perceptions.^14^, ^15^ For example, if educators are not the instigators of the innovation this may impact on their acceptance to commit to learning a new way of teaching. Also, a reluctance for educators to potentially receive poor student responses if students do not engage with the innovation.^14^ The ability of digital software to be readily updated compared to physical simulation equipment can be an advantage. It can be a disadvantage if software requires updates too often or ongoing maintenance and information technology (IT) support. Computer hardware requirements and Internet connectivity also contribute to the success or failure of implementation. It is not only the ongoing software support and maintenance, but also educators and students alike need access to ongoing support, both technical and educational, to maximise this type of learning environment for meaningful inclusion within the curricula.^15^, ^16^

### VR computed tomography (CT) in MRS education

There is limited literature on the use of VR to develop skills in CT operation. A study on student perceptions using a remote simulator for the learning of CT has recently been published. Liley et al^17^ explored MI students remotely accessing a CT scanner as part of the curriculum and highlighted challenges such as technical difficulties and a lack of facilitation as barriers to student engagement and learning. This study also emphasised the importance of when and where this learning should be placed within the curriculum. Student reports were mixed as to whether this type of learning is more beneficial prior to the CT clinical placement or to consolidate concepts after placement, or in fact both. These mixed conclusions not only highlight the need for further research but also indicate that students are individual learners and need to have opportunities which benefit their unique needs.

Bridge et al^6^ found the inclusion of the VR CT scanner simulator in the RT undergraduate programme expanded the virtual patient process from the initial scan to the final treatment.^6^ This pilot study concluded the VR RT workflow has benefits that include time‐saving, increased student confidence and optimisation of time spent at clinical placement.^6^

### CT simulation for learning

The inclusion of VR simulation in the learning and teaching of CT skills for MRS students could assist in enabling safe and repetitive practice.^6^ The use of VR simulation strategies as part of a comprehensive learning package could have the potential to better prepare students for their clinical learning. The university where this research took place does not have a physical CT scanner on‐site. Undergraduate cohorts in MI and RT are required to perform CT to meet the professional capabilities set by the Medical Radiation Practice Board of Australia.^18^ The VR CT Sim has been part of the undergraduate curriculum at (Queensland University of Technology) since 2015.

#### Medical imaging

**Figure 1:**
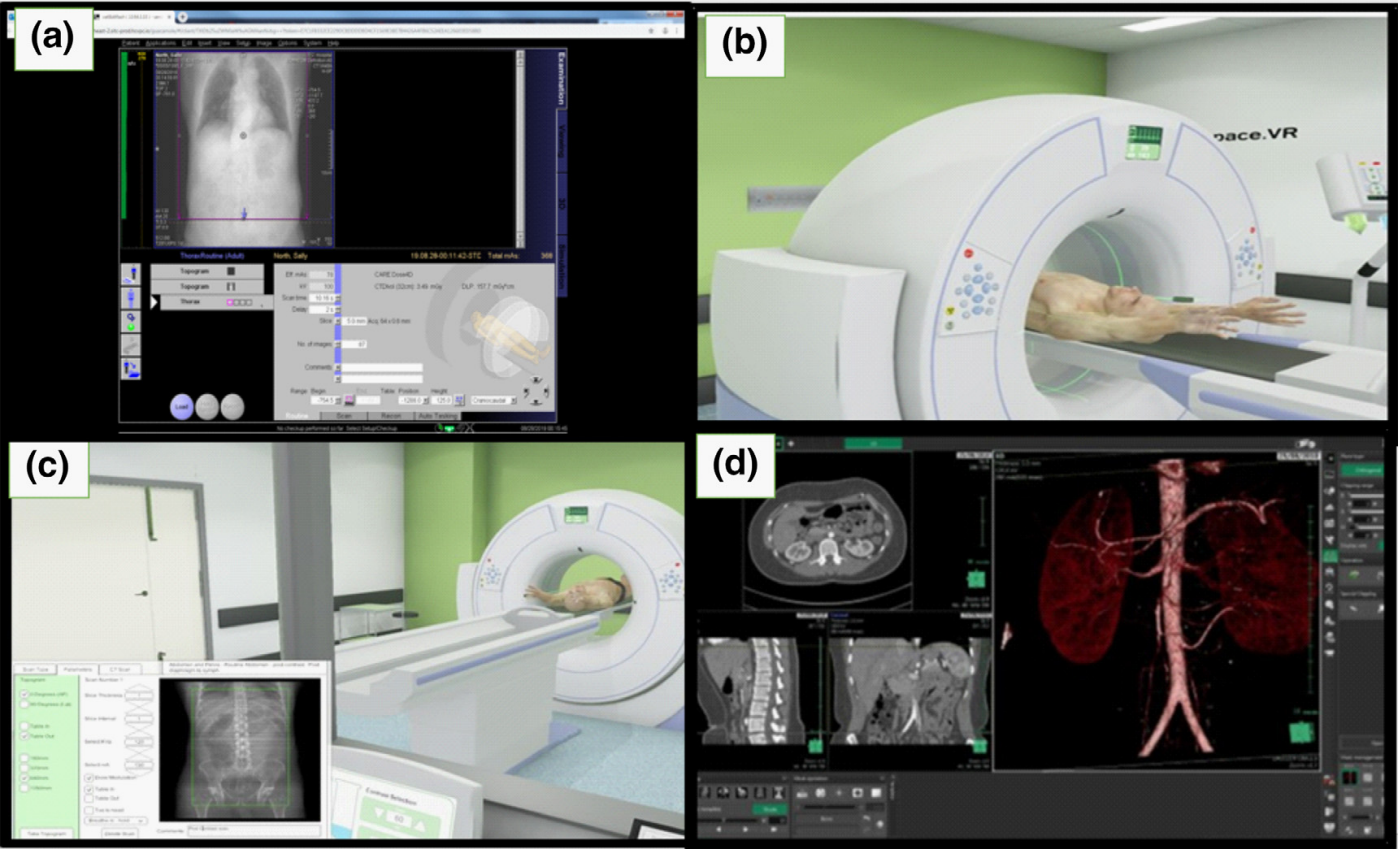
Medical Imaging VR CT simulation workflow. A: CT workstation for acquisition protocols. B: VR CT Sim for patient positioning. C: VR CT Sim for setting scan parameters. D: 3D workstation for post‐processing.

Students studying MI at Queensland University of Technology (QUT) have access to three forms of computer‐based learning for CT: a workstation simulation of acquisition protocols, and the VR CT Sim software and a three‐dimensional (3D) workstation post‐processing of data sets, see Figure 1. The workstation simulation of acquisition of protocols is utilised in instructor‐guided small group tutorials (7‐10 students). The VR CT Sim software package is accessed in instructor‐guided structured tutorials as well as for individual use for self‐directed learning. The 3D workstation post‐processing of CT data sets is undertaken in instructor‐guided structured tutorials as well as for individual use. The VR CT Sim software and 3D workstation post‐processing are available in a dedicated 30 seat computer facility. Only the simulation software package referred to as VR CT Sim was evaluated in this study. However, the authors acknowledge that VR CT Sim was a component of a wider pedagogical process and not standalone.

The CT scanner workstation (Fig. 1A) is the CT computer, which houses a working software application that diagnostic radiographers use in clinical practice. This software is not linked to a scanner and enables students to select technical protocols based on a discussed request form and then simulate a scan. This workstation is pre‐programmed with case studies and data files to provide the operator with a file of scans dependent upon the selection. There is only one workstation available during timetabled small‐group guided tutorial activities and as such is not available for individual student use and thus not included in this study.

The VR CT Sim software requires students to input the technical factors needed for the scan acquisition, replicating the process used at a clinical workstation. In addition to this, the students position the virtual patient and interact with the CT radiographic equipment which includes adjusting table height and position, location lights, with a generic gantry designed to simulate standard symbols for functionality. Students can also interact with the intravenous contrast injector and select the correct dose and dose rate as required. Students must input the appropriate acquisition delay to maximise contrast enhancement in imaging the pathology or anatomy as required. Educators pre‐load data files into the protocols to display images of contrast enhanced anatomical structures and common pathologies.

The MI picture archiving and communication system (PACS) is a repository for student radiographic images acquired during supervised practical laboratory sessions and a 3D workstation for post‐processing. It is utilised to access interesting cases and CT data sets approved for student use.^19^ The students can interact with these data sets and apply 3D reconstruction software and data analysis to CT image files.

#### Radiation therapy

**Figure 2:**
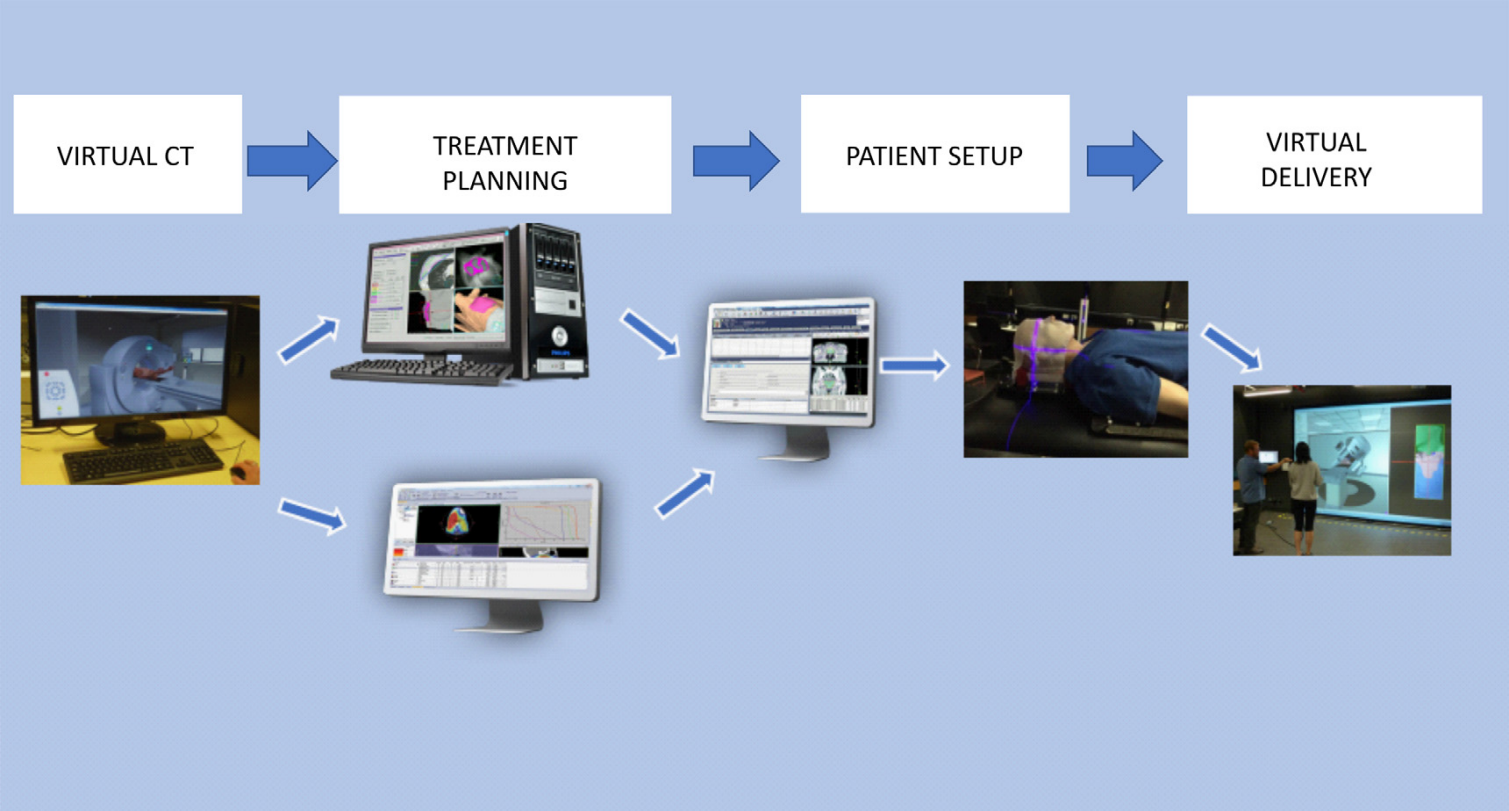
Radiation Therapy workflow used with permission Burbery^20^.

Access to the VR CT Sim software provides the RT students with the ability to perform CT simulation scans for RT planning. Students select the appropriate scanning protocols and then use the resulting pre‐loaded data sets to export to treatment planning software and ultimately the virtual treatment delivery to their RT patient, as demonstrated in Figure 2. RT students use VERT and 3D treatment planning software at university.

The first clinical component of this workflow process is the acquisition of the CT scans to inform the planning and treatment stages. Students access VR CT Sim software in the dedicated 30 seat computer facility with instructor‐led tutorial activities. The integration of VR CT Sim software enables RT students to be able to virtually practise the complete workflow.

### Research question and aim

This research aimed to investigate the impact of a computer‐based VR CT software simulation within the undergraduate curricula for MI and RT. The research question being investigated was: *What is the impact of virtual reality training on the confidence of CT scanning skill acquisition for medical imaging and radiation therapy students?*

The VR CT Sim analysis was undertaken as two separate studies due to the programme delivery for the cohorts, these being Study 1 – MI cohort – and Study 2 – RT cohort.

## Methodology

These studies aimed to measure the impact of learning via VR CT Sim on the confidence of the MI and RT undergraduate students. MI students enrolled in the 2017 second year, second semester computed tomography imaging unit, were the target participants for the MI survey. At the time of this unit of study, MI students had already experienced eight weeks of clinical placement, which may or may not have included CT. Two cohorts of RT students were the target groups for the RT study. With a change in the time of unit delivery when the degree programme changed from three years to four years, the target cohorts changed from second‐year students in 2017 to first‐year students in 2018, and thus, their clinical experience differed. A voluntary paper‐based anonymous survey using a 5‐point Likert scale with an option for student comments was available following a tutorial session. The specific methodology for each study is detailed below.

This research has been approved by the Office of Research Ethics and Integrity, QUT, under the category, Human – low risk, approval number 1400000526. As per policy and legal requirements, all data were securely stored as per ethics agreement.

### Study 1 – MI

**Figure 3:**
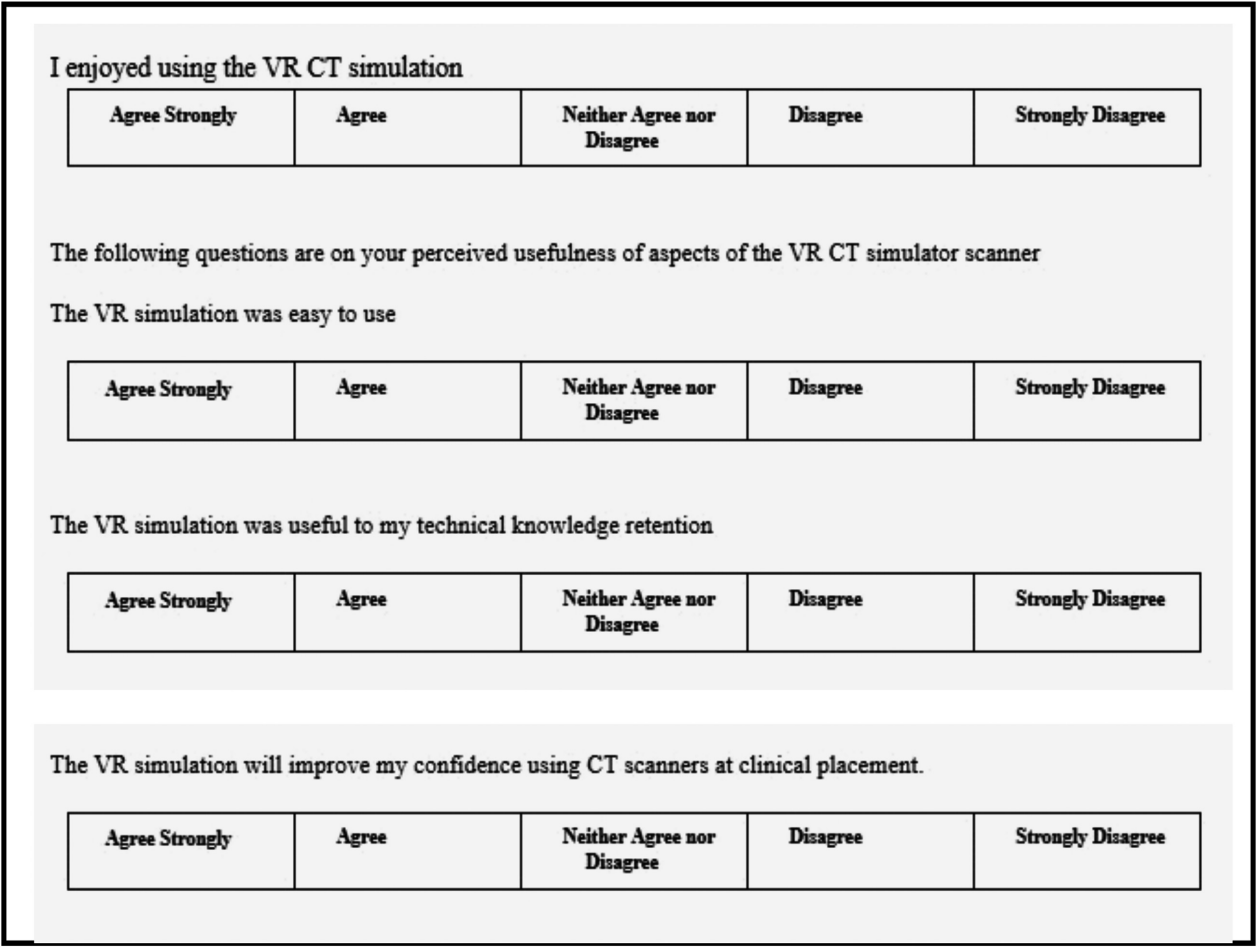
Questions from the medical imaging paper‐based survey.

Figure 3 displays questions from the MI survey form which were designed to determine whether there was an association of student engagement measures with a perceived improvement in student clinical CT skill confidence. Using SPSS version 25, analyses (numbers, frequencies for categorical variables and mean, standard deviation for continuous variables) were performed.^21^ Analyses were performed to explore an association between the student responses to usefulness, ease of use and enjoyment with the student's perceived confidence response. It was concluded that student responses of questions pertaining to the use of the VR CT Sim for ease of use, usefulness and enjoyment could provide an overall engagement indicator. The responses to these three engagement indicators were grouped into three categories and given a numeric value: 1) a negative (disagree or strongly disagree), 2) a neutral (neither agree nor disagree) and 3) a positive (agree or strongly disagree) response. These responses summed to yield a total score with a maximum of 9. A non‐parametric Fisher’s exact test was used to compare the engagement of students with the VR CT Sim and their overall perceived confidence.

### Study 2 – RT

A convenience sample of RT students within timetabled classes was asked to complete an anonymous paper survey. The RT students were provided with an introductory demonstration tutorial which later enabled them to respond via a 5 Likert‐scale survey (1 = strongly disagree to 5 = strongly agree). This survey consisted of demographic descriptors, previous clinical experiences using CT and student responses for the inclusion of VR CT Sim within the RT programme. The survey included opportunity for students to provide open‐ended comments on their experience using the VR CT Sim.

Using SPSS, frequency analysis was performed on demographic indicators (cohort, gender and age) along with analyses to explore the relationship between student confidence and demographic data, and student responses to the addition of VR CT Sim in their learning.^21^ The overall student confidence mean was calculated with standard deviation provided. The difference between cohort responses was analysed using non‐parametric Fisher’s exact tests. Student comments, although limited, were manually coded for thematic analysis using Saldaña’s three pass method.^22^

## Results

### Study 1 – MI

Of the 69 MI students enrolled in the target unit, 28 (40.6%) students completed the survey. Using a 5‐point Likert response scale (1 is strongly disagree to 5 strongly agree), students reported on their CT clinical confidence following learning activities using the VR CT Sim software resulting in a mean score of 3.39 (SD 0.737).

The number of students providing a positive response to ease of use and enjoyment of the software was evaluated at 93% and 75%, respectively. The perceived usefulness was recorded as positive by 57% of the students, where 36% responded neutrally to this statement.

When evaluating the engagement indicators, 13 students (46%) recorded either a negative or neutral response to one of the three categories of usefulness, enjoyment and/or ease of use. These students’ mean confidence score was 2.85 (95% CI 2.43, 3.26). The 15 students (54%) who agreed or strongly agreed to all engagement indicators recorded a mean confidence score of 3.87 (95% CI 3.67, 4.06). Statistical significance was demonstrated when comparing the student’s confidence levels between the groups reporting total engagement (= 9) and those less than fully engaged (<9) (Fisher’s exact test = 14.549, *P* = 0.000). The student confidence scores aligned to their engagement responses are demonstrated in Table 1.

**Table 1:** MI student mean confidence score to individual engagement categories.

Participants *n* = 28	Mean score (max = 5) for improved CT confidence	Standard Deviation
I enjoyed using the VR CT Simulation		
Disagree or strongly disagree (*n* = 1)	(2)*	
Neutral (*n* = 6)	2.7	0.816
Agree or strongly agree (*n* = 21)	3.6	0.590
The VR CT Simulation was easy to use		
Disagree or strongly disagree (*n* = 1)	(3)*	
Neutral (*n* = 2)	3	0.000
Agree or strongly agree (*n* = 25)	3.5	0.714
The VR CT Simulation was useful to my knowledge retention		
Disagree or strongly disagree (*n* = 2)	3	0.000
Neutral (*n* = 10)	2.9	0.738
Agree or strongly agree (*n* = 16)	3.8	0.577
Total engagement of VR CT Simulation – enjoyment, ease of use, knowledge retention		
Disagree/strongly disagree or neutral for 3 categories (Total score of 9>) (*n* = 13)	2.9	0.689
Agree or strongly agree for 3 categories (Total score = 9) (*n* = 15)	3.9	0.352

^*^ No mean result as *n* = 1. This was the individual confidence score for the single student.

Comments were provided by six of the 28 students (21%). Four comments demonstrated a positive response to the software, two comments related to the other simulation activities not specific to the VR CT Sim evaluated in this research, and two comments were on the structure of the learning activity using VR CT Sim. Example text comments are demonstrated in Table 2.

**Table 2:** Text excerpts for MI student comments regarding experience using VR CT Sim.

Theme	Comment
Positive	*‘It is great for positioning’* Student 1 *‘The software was helpful, and I will try (to) use the labs for study.’* Student 2 *‘Helps to get a good general idea of how CT scans work (e.g. room set up, loading contrast, patient position.) It also helps clarify and practise setting scan protocols’*. Student 3 *‘It has been useful in giving us practice prior to going on placement’*. Student 4
Learning activity	*‘I found that our tutor was unfamiliar with the software… and was unable to provide much additional help….was still great at answering our other questions!’* Student 2 *‘…exposure factors are brief and rushed’*. Student 1
Other	*‘Sim software needs to be on more than one computer (Siemens one??)’* Student 5 *‘More frequent tutorials on actual simulation, e.g. bolus tracking rather than just post‐processing’*. Student 6

### Study 2 – RT

Of the 81 students enrolled in the target cohorts, 38 (47%) completed surveys and of these 28 were under 21 years of age.

Overall, the mean score for student responses to feeling confident in their abilities to perform a CT planning scan at their next clinical placement was 2.61 (SD 0.916), on the 5‐point Likert scale. A Fisher’s exact test was conducted to compare mean confidence scores between the 2017 and 2018 cohorts. Statistical significance was not demonstrated (Fisher’s exact test = 5.609, *P* = 0.060). Table 3 demonstrates the demographic breakdown and student mean CT confidence scores.

**Table 3:** Breakdown of RT CT student participation demographics and subsequent mean CT confidence score.

Demographics	Participant numbers (Total = 38)	Mean score (max = 5) for CT confidence at the next clinical placement
Age		
Under 21	28 (74%)	2.61 (±0.916)
21 and over	10 (26%)	2.63 (±1.061)
Gender		
Male	9 (24%)	2.67 (±1.000)
Female	27 (71%)	2.63 (±0.926)
Other or none	2 (5%)	

Cohort	Enrolled students	Participants	
2017 – 2^nd^‐year students	42	18 (43%)	3.00 (±0.907)
2018 – 1^st^‐year students	39	20 (52%)	2.25 (±0.786)
Total	81	38 (47%)	2.61 (±0.916)

**Figure 4:**
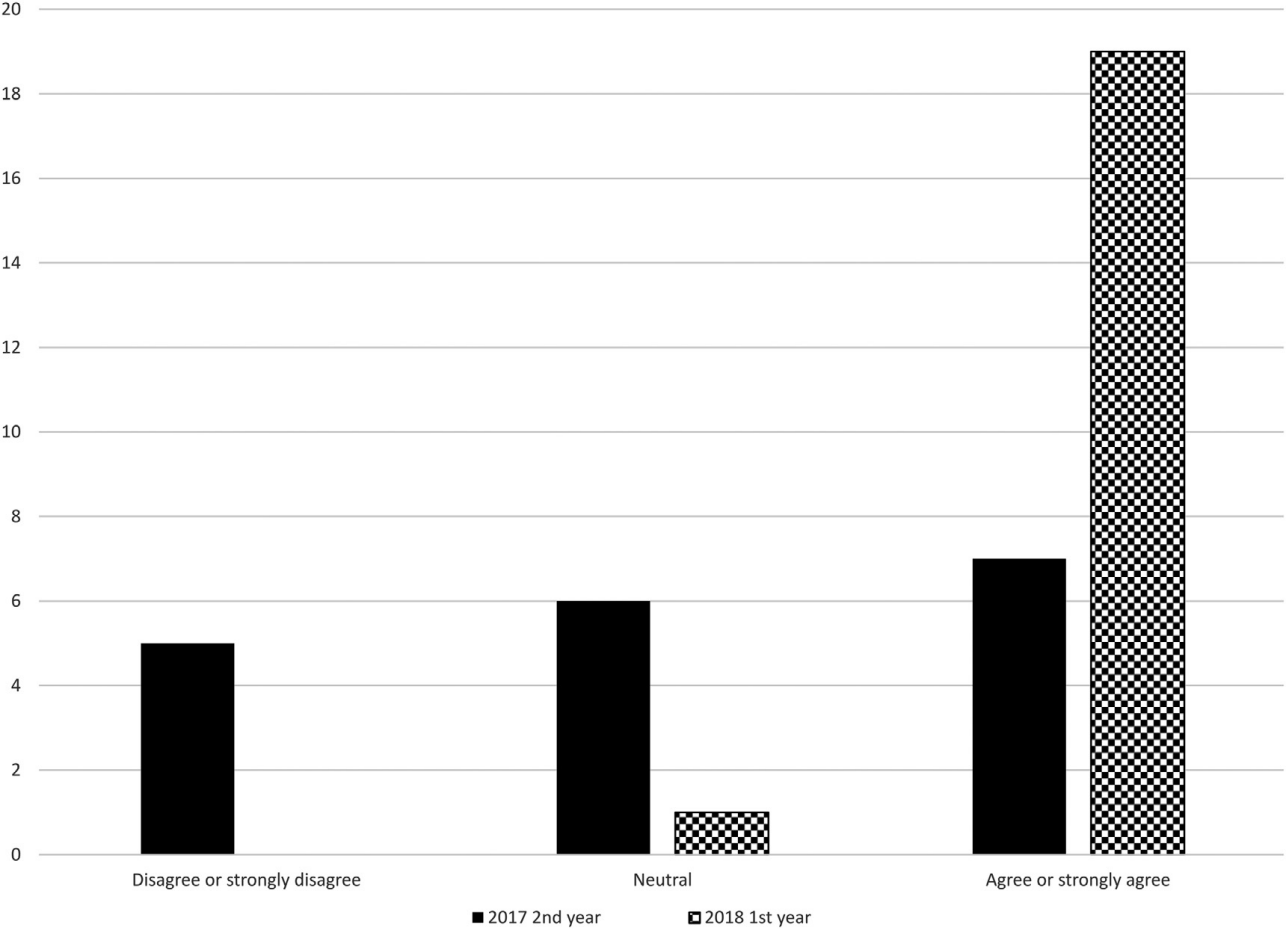
RT student response to ‘the inclusion of the CT scanner and acquiring the images helps me learn the process of my role as a radiation therapist better’.

Student responses to the statement ‘*The inclusion of the CT scanner and acquiring the images helps me learn the process of my role as a radiation therapist better*’ yielded 68% of students either agreed or strongly agreed to this statement. Figure 4 demonstrates this distribution for each cohort, with 95% of the 2018 first‐year cohort agreeing or strongly agreeing to the inclusion of VR CT simulation as being helpful to their learning, this yielded a statistical significance (Fisher’s exact test = 15.6, *P* = 0.001).

Open‐ended comment responses were provided by 13 students, 34% of participants. Three themes arose, positive responses, negative responses including limited exposure as a category, and clinical placement. Table 4 highlights some examples of the comments and themes provided by students from each cohort.

**Table 4:** Text excerpts for RT student comments regarding experience using VR CT Sim.

Theme	Cohort	Student Comment
Positive	2017 (2^nd^ year)	‘*Overall it was a good experience*’. Student 1
	2018 (1^st^ year)	‘*Great for understanding concepts in low pressure environment’*. Student 4 *‘Has allowed me to familiarise with the environment before proceeding with placement where everyone was extremely nervous. So having that opportunity did lesson worries’*. Student 5
Negative	2017 (2^nd^ year)	*‘I have only used (CT VR Sim) a couple of times. I did not feel like it assisted me at all. Clinical placement is a much better learning opportunity for me personally*’. Student 2
Clinical Placement	2017 (1^st^ year)	*‘I would prefer to use a CT machine on placement as my main method of learning*’. Student 3

## Discussion

### MI

Students’ mean improved confidence was reported at being above neutral on the 5‐point Likert scale for learning using the VR CT Sim software. Whilst not being a conclusive result, the implication that the use of VR CT Sim in learning is of no disadvantage to the students’ confidence is promising.

The positive results of the students’ ease of use and enjoyment of the software could be explained by these students’ familiarity with the software menus due to using a linked general radiography VR module in their previous two semesters of study. The group of students that agreed or strongly agreed with all three engagement factors had a reported mean confidence score 1.02 higher than those that remained neutral or disagreed with either of these factors. It could be concluded that these students’ engagement in this learning opportunity enabled their perceived confidence in performing this skill clinically to be higher than those that were not completely engaged.

The research question aimed to measure the impact of learning via VR CT Sim on student confidence. The positive comments of the students valuing the additional process learning within VR CT Sim are highlighted in the comment ‘(VR CT Sim) *helps to get a good general idea of how CT scans work (e.g. room set up, loading contrast, patient position.) It also helps clarify and practise setting scan protocols*’. One highlighted advantage of using VR simulation in the literature is the ability for the software to be used for self‐directed learning^15^; however, student comments indicated a need for educator expertise in the use of the software program.

### RT

The overall confidence of the RT students in their abilities to perform a CT scan at their next clinical placement was above average. No significant difference was demonstrated when comparing the cohort responses to reporting confidence in performing CT on the next clinical placement. However, there was a significant difference in the responses of the 2018 students to the positive inclusion the VR CT Sim had on their learning of acquiring CT within the RT process. This could be attributed to the 2018 first‐year cohort not having the 7 weeks of clinical experience in CT compared with those in the second‐year cohort of 2017.

The qualitative results once again reinforce the benefits of VR simulation aligning with the known advantages within the wider literature.^12^, ^13^, ^14^ Students highlighted the preference to learn CT in the clinical environment; however, some knowledge prior to this to enable ‘familiarisation’ as stated in the student comments could have the potential to improve student placement efficiencies. Currently, computer simulation is used widely within the RT programme for planning and treatment delivery.

### Combined discussions

The use of this innovative integration of VR within MRS education is promising. Whilst these results are not without limitations, the perceived confidence of students entering clinical placement is important not only to enhance the student experience but to see a flow on effect of student efficiencies in the clinical environment.

Students have indicated a need for guided and supervised practice when using the VR CT Sim whilst concurrently highlighting that one of its advantages is for self‐directed learning. This demonstrates the importance to recognise individual student needs and to provide varied learning opportunities for them to engage with. The studies reported here relate to multiple access tutorials for MI and limited access for RT. Despite these differences, the VR CT Sim activity was facilitated and structured for both groups.

### Limitations

The results presented in this study refer to student perceived confidence. This is a subjective measure and needs to be assessed in the knowledge that personal, behavioural and environmental determinants are all factors for general confidence,^23^ as well as the learning activities assessed within this research. The ability of the student to consciously separate these other components is difficult to distinguish. The original MI research design included a pre‐ and post‐comparative survey including a knowledge skill test. However, comparative responses were limited and thus were not part of this analysis.

The course structure during the timeframe of this research was being modified and transitioned from a three‐year to a four‐year programme. In making comparisons between the cohorts, it is necessary to be mindful of the change of RT participants from second‐year to first‐year students.

The authors acknowledge the RT students had limited engagement opportunities with the VR CT Sim software, and as such, results need to be approached as an overall response to the context of simulated learning.

## Conclusions

This study aimed to measure MRS student confidence in performing a CT scan in the clinical environment after using VR CT Sim as a learning tool. The overall result from both studies was that students agreed that access to this simulation was beneficial to their clinical CT confidence. The importance of safe practice in a low‐pressure environment as reported in the literature is all critical findings that can have the potential to improve clinical confidence.^13^, ^14^, ^15^

Further studies are required to investigate whether the inclusion of VR CT Sim improves student technical skill knowledge; however, this study has indicated there is potential for students to gain time‐learning CT skills in a safe, low‐pressure, structured and accessible VR environment. Whilst this study looked at one aspect of the pedagogical approach to structured CT skill acquisition, the results show this approach could be further explored to provide simulation to supplement CT learning. Emerging global challenges due to the COVID‐19 pandemic highlight the significance of simulation to support student learning.

## Conflict of Interest

The lead author wishes to declare directorship of the CT VR simulation software discussed in the paper.
